# Computational Evaluation and Multi-Criteria Optimization of Natural Compound Analogs Targeting SARS-CoV-2 Proteases

**DOI:** 10.3390/cimb47070577

**Published:** 2025-07-21

**Authors:** Paul Andrei Negru, Andrei-Flavius Radu, Ada Radu, Delia Mirela Tit, Gabriela Bungau

**Affiliations:** 1 Doctoral School of Biological and Biomedical Sciences, University of Oradea, 410087 Oradea, Romania; negru.paulandrei@student.uoradea.ro (P.A.N.); dtit@uoradea.ro (D.M.T.); gbungau@uoradea.ro (G.B.); 2 Department of Preclinical Disciplines, Faculty of Medicine and Pharmacy, University of Oradea, 410073 Oradea, Romania; 3 Department of Psycho-Neurosciences and Recovery, Faculty of Medicine and Pharmacy, University of Oradea, 410073 Oradea, Romania; 4 Department of Pharmacy, Faculty of Medicine and Pharmacy, University of Oradea, 410028 Oradea, Romania

**Keywords:** COVID-19, in silico, SARS-CoV-2, molecular docking, virtual screening, 3Clpro, PLpro, *Zingiber officinale*, *Allium sativum*

## Abstract

The global impact of the COVID-19 crisis has underscored the need for novel therapeutic candidates capable of efficiently targeting essential viral proteins. Existing therapeutic strategies continue to encounter limitations such as reduced efficacy against emerging variants, safety concerns, and suboptimal pharmacodynamics, which emphasize the potential of natural-origin compounds as supportive agents with immunomodulatory, anti-inflammatory, and antioxidant benefits. The present study significantly advances prior molecular docking research through comprehensive virtual screening of structurally related analogs derived from antiviral phytochemicals. These compounds were evaluated specifically against the SARS-CoV-2 main protease (3CLpro) and papain-like protease (PLpro). Utilizing chemical similarity algorithms via the ChEMBL database, over 600 candidate molecules were retrieved and subjected to automated docking, interaction pattern analysis, and comprehensive ADMET profiling. Several analogs showed enhanced binding scores relative to their parent scaffolds, with CHEMBL1720210 (a shogaol-derived analog) demonstrating strong interaction with PLpro (−9.34 kcal/mol), and CHEMBL1495225 (a 6-gingerol derivative) showing high affinity for 3CLpro (−8.04 kcal/mol). Molecular interaction analysis revealed that CHEMBL1720210 forms hydrogen bonds with key PLpro residues including GLY163, LEU162, GLN269, TYR265, and TYR273, complemented by hydrophobic interactions with TYR268 and PRO248. CHEMBL1495225 establishes multiple hydrogen bonds with the 3CLpro residues ASP197, ARG131, TYR239, LEU272, and GLY195, along with hydrophobic contacts with LEU287. Gene expression predictions via DIGEP-Pred indicated that the top-ranked compounds could influence biological pathways linked to inflammation and oxidative stress, processes implicated in COVID-19’s pathology. Notably, CHEMBL4069090 emerged as a lead compound with favorable drug-likeness and predicted binding to PLpro. Overall, the applied in silico framework facilitated the rational prioritization of bioactive analogs with promising pharmacological profiles, supporting their advancement toward experimental validation and therapeutic exploration against SARS-CoV-2.

## 1. Introduction

Coronavirus disease 2019 (COVID-19) is a viral condition triggered by the recently identified single-stranded RNA virus called severe acute respiratory syndrome coronavirus 2 (SARS-CoV-2) [1], a member of the Coronaviridae family, which is taxonomically classified into the four genera, known as *Alphacoronavirus*, *Betacoronavirus*, *Gammacoronavirus*, and *Deltacoronavirus* [2]. Initially identified as a respiratory condition in Wuhan, China, toward the close of 2019, this viral outbreak swiftly escalated into a global pandemic, posing an unprecedented challenge to contemporary public health systems. Currently, COVID-19 continues to represent a global health issue, primarily due to the persistent evolution of SARS-CoV-2 and its adaptive evolution in certain geographic regions [3,4]. As of April 2025, a total of 778 million COVID-19 cases have been reported worldwide, with the United States having the highest cumulative total at 103 million cases, followed by China with 99.4 million, India at 45 million, France with 39 million, and Germany at 38.4 million, according to the World Health Organization [5].

The mechanisms underlying SARS-CoV-2 infection are multi-faceted, primarily involving disruptions in immune function and endothelial cell impairment. By binding to the angiotensin-converting enzyme 2 (ACE2) receptor, the virus activates immune pathways that release inflammatory mediators such as tumor necrosis factor, initiating a cascade of cytokines and inducing tissue harm. The dysfunction of lymphocytes—notably, the decline in T-cell counts—further worsens the clinical outcome. In addition, the virus exerts a direct impact on endothelial cells, heightening vascular permeability and promoting the formation of blood clots, which can lead to severe complications, including respiratory failure and heart injury [6,7]. The management of SARS-CoV-2 infection required the evaluation of several drug categories through extensive clinical trials, with a focus on antiviral therapies such as small-molecule inhibitors and monoclonal antibodies. These treatments were designed to interfere with critical steps in the viral replication cycle, or to directly neutralize the virus [8].

To address the uncertainties related to SARS-CoV-2, the approach of repurposing existing drugs was pursued, evaluating treatments such as chloroquine and hydroxychloroquine (i.e., antimalarial), ivermectin (i.e., antiparasitic), and ebselen (i.e., anti-inflammatory), alongside antiviral agents like remdesivir, molnupiravir, and favipiravir, which are nucleoside analogs. Additionally, monoclonal antibodies, including bebtelovimab, sotrovimab, and crizanlizumab, were investigated. However, only a small number of the initial options have been granted approval for clinical use in managing SARS-CoV-2 infection [9]. Pharmacological agents authorized or approved by the US Food and Drug Administration for the treatment of SARS-CoV-2 infection, such as remdesivir, molnupiravir, nirmatrelvir/ritonavir, and monoclonal antibodies like sotrovimab and casirivimab/imdevimab, exhibit varying levels of efficacy and benefits in shortening hospitalization or reducing the severity of illness. In contrast, drugs such as ribavirin, galidesivir, ivermectin, lopinavir/ritonavir, and favipiravir have either not received approval or are subject to limited indications, with many showing suboptimal effectiveness or yielding inconsistent results in clinical trials, while some may also be associated with side effects such as nausea or diarrhea [10].

Given the complexity of the pathophysiological mechanism, the diversity of therapies targeting different factors, the fact that only a few of the tested and repurposed drugs have been approved, and the impact of viral mutations, comprehensive in silico studies are essential for identifying and testing new compounds, as well as for advancing research on the discovery of therapeutic targets.

There is an approximate 80% genetic similarity between SARS-CoV-2 and SARS-CoV, both displaying considerable parallels in their protein architecture and functionality. Among the key proteases in SARS-CoV-2, the 3-chymotrypsin-like protease (3CLpro) and papain-like protease (PLpro) are instrumental in processing the viral polyproteins derived from the RNA genome [11]. After the virus infiltrates the host cell, it undergoes disassembly, releasing the nucleocapsid along with the viral genome. The host’s ribosomes then translate open reading frame (ORF) 1a/b, which results in the production of two polyproteins, pp1a and pp1ab, encoding 16 non-structural proteins (nsps). The remaining ORFs are responsible for encoding structural and accessory proteins. The cleavage of the polyproteins is carried out by two key proteases: the main protease (3CLpro, nsp5) and the papain-like protease (PLpro, nsp3). These proteases generate nsps 2 through 16, which are essential components of the replication–transcription complex [12]. This processing is essential for the generation of functional proteins that are critical for viral replication and propagation. Inhibition of these enzymes could significantly impair the virus’s ability to replicate, thereby reducing the transmission rate. The mechanistic overlap in how SARS-CoV and SARS-CoV-2 infect host cells has greatly informed therapeutic research [11,13,14]. As these proteases are central to the viral replication cycle, they represent promising targets for in silico methodologies, including molecular docking and virtual screening, to identify new potential therapeutics for SARS-CoV-2.

In the light of the unmet needs of conventional therapies, natural sources offer a rich reservoir of bioactive compounds that can contribute to the development of new drug candidates, serving as frameworks for the design of novel therapeutic agents. During health emergencies, such as the COVID-19 pandemic, where no effective treatment was initially available, these natural compounds can provide valuable insights into potential therapeutic approaches for disease management and prevention [15]. The anti-inflammatory and antioxidant effects demonstrated by some bioactive compounds have served as the basis for numerous promising in silico studies targeting complex pathologies [16,17]. The search for efficient and cost-effective strategies to discover novel and adjuvant treatments against coronaviruses is still a priority. Virtual screening, combined with molecular docking, represents in silico tools using computational models to evaluate the interactions between candidate compounds and specific molecular targets. Natural compounds offer a promising pool of molecules for such screenings, with their vast array of chemical structures, many of which are already recognized for their proven therapeutic benefits [18].

While numerous computational studies have investigated SARS-CoV-2 protease inhibitors, most have focused on either random compound library screening or repurposing existing drugs, often evaluating compounds against single targets without comprehensive safety and pharmacological assessments. The present study addresses these limitations through a systematic approach that combines strategic expansion of natural product scaffolds with demonstrated clinical benefits in COVID-19 patients, comprehensive evaluation against multiple viral targets to identify dual-inhibitory compounds, integration of advanced toxicity prediction and gene expression analysis to assess potential immunomodulatory effects, and rigorous ADMET profiling to prioritize compounds with favorable drug-like properties. This multi-faceted approach provides a more holistic framework for identifying therapeutically viable candidates compared to studies focusing solely on binding affinity.

Previous research has demonstrated that a variety of natural compounds and their synthetic analogs, identified through computational biology screening and biological in vitro assays, show promising inhibitory effects against several key proteases involved in the replication and progression of SARS-CoV-2. These proteases include Mpro (3CL-protease), RNA-dependent RNA polymerase, cathepsin-L, TMPRSS2, Sec61, and the cap-binding proteases eIF4A, eIF4E, and eEF1A, all of which play pivotal roles in viral activity within host cells [19].

Among plant-derived therapeutic candidates, *Zingiber officinale* has emerged as a notable source of bioactive compounds with promising pharmacological effects. Several of its constituents, such as gingerol, shogaols, paradol, and zingerone, have been extensively documented for their capacity to exert antioxidant and anti-inflammatory effects, as well as for their potential immunomodulatory action. A substantial body of evidence highlights *Zingiber officinale*’s wide-ranging biological properties, particularly its ability to modulate oxidative stress and inflammatory responses [20,21].

Alongside *Zingiber officinale*, *Allium sativum* L. represents another extensively utilized botanical with significant medicinal potential, particularly due to its richness in organosulfur compounds. This commonly used culinary plant harbors a diverse array of biologically active molecules, including ajoene, S-allyl cysteine, diallyl trisulfide, allicin, diallyl disulfide, alliin, and diallyl sulfide. Numerous investigations have consistently supported the therapeutic relevance of these constituents, attributing to them potent immune-regulating, anti-inflammatory, and antioxidant properties [22].

Given the pivotal involvement of the cysteine proteases 3CLpro and PLpro in both the replication cycle of SARS-CoV-2 and its strategies to evade host immunity, the investigation of natural bioactive substances with established anti-inflammatory and antioxidant functions, such as those isolated, tested, and validated from *Zingiber officinale* and *Allium sativum* L., is scientifically justified. Preliminary in silico screening of several known bioactive molecules derived from *Zingiber officinale* and *Allium sativum* L, selected for their traditional therapeutic use and established pharmacological properties, has revealed promising binding affinities against SARS-CoV-2 proteases, namely, the main protease 3CLpro and PLpro. These prior evaluations revealed that natural compounds such as shogaol, 6-gingerol, and S-allyl-cysteine demonstrated noteworthy binding affinities for 3CLpro, while shogaol, 6-gingerol, and 8-gingerol showed favorable predicted interactions with PLpro [23].

The present study aims to strategically integrate a multi-phase in silico drug discovery framework targeting SARS-CoV-2 proteases, by expanding validated leads through fingerprint type 2 (FP2) screening of the ChEMBL database. The subsequent virtual screening against both 3CLpro and PLpro employs validated molecular docking protocols to identify the candidates with the highest potential for interaction. The integration of rigorous binding mode characterization, ADMET profiling, and mRNA-based prediction results provides a methodological advantage for identifying candidates that exhibit not only superior protease affinity but also essential drug-like characteristics.

## 2. Materials and Methods

### 2.1. Ligand Dataset Preparation

A set of lead compounds previously identified for their promising binding affinities to key SARS-CoV-2 proteases [23] were selected as the basis for similarity searches to identify structurally related analogs. These compounds were derived from *Zingiber officinale* and *Allium sativum* L., selected for their traditional therapeutic applications and documented use as supplements during the COVID-19 pandemic. Both plants have demonstrated promising antiviral potential against SARS-CoV-2 through multiple mechanisms. Clinical studies have shown that the use of garlic extracts as adjuvant therapy reduced oxygen requirements in COVID-19 patients, while ginger supplementation significantly shortened hospitalization time, particularly in vulnerable populations [24,25].

Molecular docking studies have identified that bioactive compounds in both plants, including gingerols and shogaols from ginger and organosulfur compounds from garlic, can bind to SARS-CoV-2 proteases. Additionally, these natural products offer anti-inflammatory, antioxidant, and immunomodulatory properties that may help mitigate the cytokine storms associated with severe COVID-19, with the added advantage of favorable safety profiles due to their long history of human consumption as food sources [26,27].

For 3CLpro, we selected shogaol, 6-gingerol, and S-allyl-cysteine, with docking scores of −5.9, −5.9, and −5.5 kcal/mol, respectively. In the case of PLpro, we chose shogaol, 6-gingerol, and 8-gingerol based on their favorable interaction profiles, showing binding energies of −7.6, −6.7, and −6.6 kcal/mol, respectively. These parent molecules were used to generate a library of structurally similar analogs for further computational screening.

### 2.2. Similarity Search Methodology

The Swiss similarity server (http://www.swisssimilarity.ch/, accessed on 13 January 2025) was used to identify compounds with similar structures to the parent molecules. This research exclusively employed the FP2 methodology to identify structural analogs of the parent compounds. In this method, molecules up to seven atoms long can be detected and recorded by detecting their linear and branched molecular fragments. Such characteristics make the FP2 approach particularly effective for discovering small molecules with comparable structural properties that may exhibit bioactive potential against the intended protease targets.

For each parent compound, similarity searches were conducted exclusively in the ChEMBL database of bioactive compounds (complete database). This targeted strategy was adopted to increase the likelihood of finding compounds with advantageous pharmacological characteristics by ensuring that all discovered analogs already had proven bioactivity data. The RDKit toolkit preserved stereochemical information from the input SMILES during the 3D structure generation process, ensuring that the specific stereoisomeric forms retrieved from ChEMBL were accurately represented in the molecular docking simulations. Therefore, all reported binding affinities correspond to specific enantiomers or diastereomers, as defined in the original ChEMBL entries. To make sure that only compounds that share enough structural similarities with their parent molecules were kept for additional examination, a similarity criterion of 0.7 (Tanimoto coefficient) was set. Compound identifiers, simplified molecular input line entry system (SMILES) notations, and matching similarity scores for each analog in relation to its parent compound were included in the CSV files that were exported from the search results [28]. It is important to note that the ChEMBL database entries include stereochemical information when available, with SMILES notations containing stereochemical descriptors. This ensures that each retrieved compound represents a specific stereoisomeric form. The preservation of this stereochemical information was maintained throughout our computational workflow.

### 2.3. Preparation of Compounds for Molecular Docking

**Figure 1:**
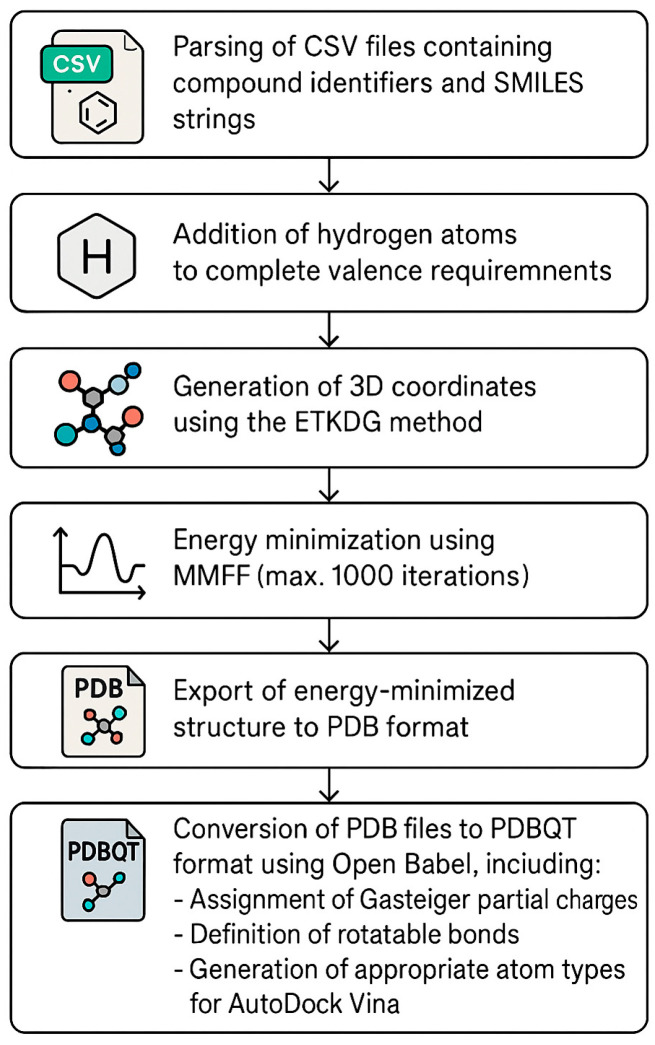
Automated ligand preparation pipeline for high-throughput virtual screening, integrating RDKit and Open Babel for the conversion of SMILES to docking-ready structures. CSV, comma-separated values; SMILES, simplified molecular input line entry system; ETKDG, experimental-torsion knowledge distance geometry; MMFF, Merck molecular force field; PDB, Protein Data Bank; PDBQT, Protein Data Bank, partial charge (q), and atom type (t) format.

Given the extensive number of structural analogs retrieved through the similarity searches, a streamlined and automated processing workflow was implemented to ensure efficiency and consistency in compound preparation. To address this challenge, a custom Python version 3.12.3 script was developed to automate the conversion of SMILES notations to three-dimensional structures suitable for AutoDock Vina version 1.2.0. docking. This script leveraged two major cheminformatics toolkits: RDKit version 2025.3.3 for initial 3D structure generation and conformer optimization, and Open Babel version 3.1.1, for final conversion to PDBQT format (https://www.rdkit.org, accessed on 12 January 2025). The preparation workflow is presented in Figure 1.

The Python script utilized the following command to convert the optimized PDB structures to PDBQT format: obabel {pdb_file} -O {pdbqt_file} -xh --partialcharge Gasteiger.

After processing, the final ligand dataset consisted of 600 unique compounds derived from the parent molecules, with each compound represented as a PDBQT file containing 3D coordinates, partial charges, atom types, and rotatable bond definitions required for molecular docking simulations.

### 2.4. Target Protein Preparation

The crystal structure of the two proteins of interest (i.e., 3CLpro with the PDB ID: 7EN9, and PLpro with the PDB ID: 7TZJ) was obtained as .pdb files from the Protein Data Bank. These two proteases are essential for the processing of viral polyproteins into functional non-structural proteins required for viral replication. 3CLpro cleaves at 11 polyprotein sites to release itself and other nsps, including the RNA-dependent RNA polymerase, while PLpro cleaves at three sites within the polyprotein to release nsps 1–3 [29].

The 3CLpro active site is comprised of a catalytic dyad consisting of His41 and Cys145, which are essential for proteolytic activity through a nucleophilic attack mechanism. The substrate-binding site is organized into multiple subsites (S1′, S1, S2, and S4), which accommodate the P1′, P1, P2, and P4 residues of the substrate, respectively. The S1 subsite, which confers substrate-specificity, is formed by His163, Glu166, Phe140, and Leu141, providing a strong preference for glutamine at the P1 position. The S2 subsite is characterized by its predominance of hydrophobic properties, encompassing Met49, Met165, and His41. In contrast, the S4 subsite is distinguished by the presence of Met165, Leu167, Pro168, and Gln189. It is notable that additional structurally significant residues include Thr26 and Thr25 at the binding pocket entrance; Arg188, which is instrumental in anchoring substrates; and Gln192, which plays a crucial role in substrate recognition [30,31].

The PLpro active site features a catalytic triad composed of Cys111, His272, and Asp286, employing a papain-like cysteine protease mechanism. The substrate-binding region is characterized by a palm–thumb–fingers architecture typical of USP-family deubiquitinases. Key residues forming the substrate-binding cleft include Asp164, which is critical for both protease and deubiquitinase activities, and the BL2 loop residues (Tyr264, Tyr268, Tyr273) that undergo conformational changes upon substrate binding. The substrate-binding channel is partially formed by Gly163 and Leu162, while Pro247 and Pro248 contribute to the structural framework of the binding pocket. Thr301 plays a crucial role in substrate recognition, and Gln269 participates in stabilizing substrate interactions. The zinc-binding domain, coordinated by Cys189, Cys192, Cys224, and Cys226, provides structural stability to the thumb subdomain [32].

The protein preparation process is a critical step in the overall procedure, and it is necessary to ensure that the conditions for molecular docking are optimal. It is imperative to note that water molecules were removed in order to avoid interference with ligand–protein interactions during the docking simulation. Similarly, all heteroatoms, including co-crystallized ligands, were eliminated from the structures. This cleaning process was performed using Molegro Molecular Viewer version 2.5. The prepared protein structures were saved in PDBQT format, which includes the partial charges and atom types necessary for docking simulations with AutoDock Vina, as described in Section 2.5.

### 2.5. Molecular Docking Setup and Virtual Screening Protocol

The molecular docking of the prepared ligands against SARS-CoV-2 proteases was performed using AutoDock Vina version 1.2.5. For both target proteins, custom configuration files were created to define the search space and docking parameters. For 3CLpro, the grid box was centered at the coordinates X = 15.94, Y = 26.70, Z = −18.19, while for PLpro, the grid box was centered at X = −2.69, Y = 4.74, Z = −35.21. Both grid boxes had dimensions of 60 Å × 60 Å × 60 Å to fully encompass the respective binding sites and allow sufficient space for ligand conformational sampling [33,34].

The number of binding modes to generate was set to nine, the energy range was set to 3 kcal/mol to capture all biologically relevant binding poses, and exhaustiveness was set to eight to balance search thoroughness with computational efficiency. These docking parameters were standardized across all simulations to ensure comparability of the results. These parameters were validated through re-docking of the respective co-crystallized ligands (WU-02 for 3CLpro and inhibitor 3k for PLpro) into their binding sites. The binding modes of selected top-scoring compounds were analyzed using BIOVIA Discovery Studio Visualizer 2025 (Dassault Systèmes, San Diego, CA, USA).

**Figure 2:**
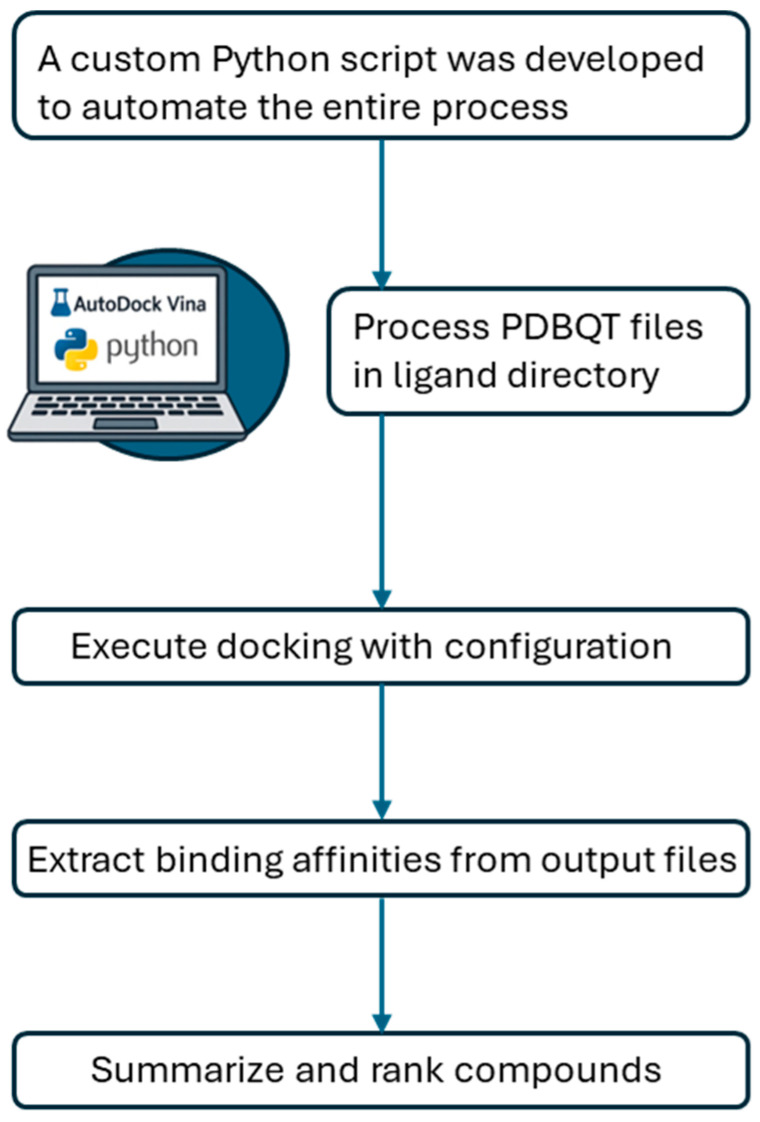
Automated virtual screening workflow.

For the virtual screening campaign, a custom Python script was developed to automate the entire process. This script implemented a DockingRunner class that handled batch processing of the large compound libraries, submitting each ligand for docking against both protease targets. The script performed several key functions: verification of AutoDock Vina installation, systematic processing of all PDBQT files in the ligand directory, execution of docking with the appropriate configuration, extraction of binding affinities from output files, and compilation of the results into a comprehensive summary file. The compounds were then ranked based on their binding affinities, with more negative values indicating stronger predicted binding. Figure 2 summarizes the steps of the process.

### 2.6. ADME Characteristics Prediction

The physicochemical properties and absorption, distribution, metabolism, and excretion (ADME) profiles of the top-performing compounds were comprehensively evaluated using the SwissADME web-based computational platform (http://www.swissadme.ch/, accessed on 27 January 2025). The structural information for each identified compound, formatted as standardized SMILES notations, was analyzed using the SwissADME computational platform. This system applies validated predictive algorithms to calculate a diverse array of pharmacokinetic characteristics.

### 2.7. Toxicity Risk Assessment

ProTox-III, an online program that employs a thorough approach to toxicity prediction, was used to analyze the toxicity risk for the top-performing chemicals. ProTox-III predicts a variety of toxicological endpoints by combining chemical similarity, fragment-based techniques, and machine learning algorithms [35]. Two primary areas were the focus of the analysis after the chosen compounds’ SMILES notations were uploaded to the ProTox-III server: organ toxicity predictions, and toxicity endpoint predictions.

### 2.8. mRNA-Based Prediction Results

Drug-induced gene expression profile prediction (DIGEP-Pred) is a web-based tool designed to predict how the chemical structure of a compound may influence gene expression changes induced by drug exposure [36]. The prediction is based on structure–activity relationships generated using the PASS-2024 software. To train its models, DIGEP-Pred uses both mRNA- and protein-based datasets derived from the Comparative Toxicogenomics Database (https://ctdbase.org/, accessed on 28 January 2025), which contains curated information on known drug-induced gene expression profiles.

A probability threshold above 0.7 was selected to ensure high-confidence gene expression predictions, minimizing false positives and enhancing biological relevance in downstream analyses.

Table 1 provides a summary of the tools used to evaluate the overall execution of this study.

## 3. Results and Discussion

### 3.1. Validation of the Docking Protocol

The implementation of an automated high-throughput virtual screening methodology required validation of the docking protocol in order to ensure that the combination of automated ligand preparation and batch docking procedures maintained the accuracy of the binding pose predictions.

The reliability of the automated docking methodology was assessed by re-docking the co-crystallized inhibitors into their respective protein-binding sites. For 3Clpro (PDB ID: 7EN9), the native ligand, WU-02, was re-docked, resulting in an RMSD of 0.933 Å between the crystallographic and predicted poses. The highest-scoring pose exhibited a binding affinity of −7.61 kcal/mol, with the nine generated conformations ranging from −7.61 to −6.63 kcal/mol.

Similarly, for PLpro (PDB ID: 7TZJ), the re-docking of inhibitor 3k yielded an RMSD of 0.968 Å for the top-scoring pose, which had a binding affinity of −9.58 kcal/mol. The binding modes ranged from −9.58 to −8.14 kcal/mol.

**Figure 3:**
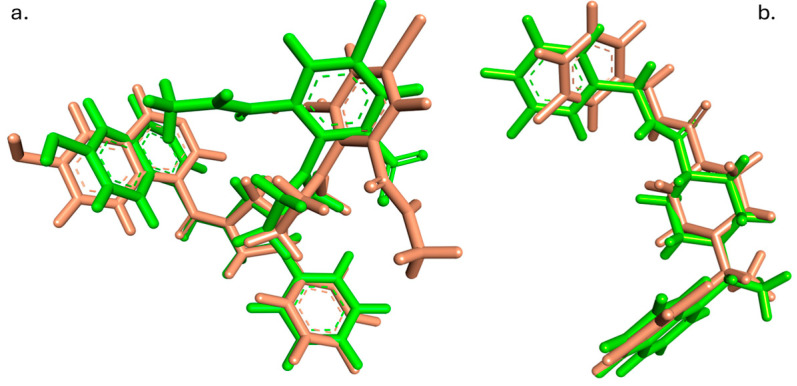
Validation of docking protocol accuracy demonstrated by superimposition of crystallographic (green) and computationally docked (brown) conformations of (**a**) WU-02 in the 3CLpro-binding pocket and (**b**) inhibitor 3k in the PLpro-binding pocket.

Both validation results yielded RMSD values below the generally accepted threshold of 2.0 Å, confirming that the automated docking protocol could accurately reproduce the experimentally determined binding modes despite the transition to a high-throughput computational pipeline. These RMSD values demonstrated that the integration of RDKit-based ligand preparation with AutoDock Vina in an automated workflow did not compromise the accuracy of the binding pose predictions, thereby providing confidence in the reliability of the subsequent virtual screening results. The superimposition of the docked poses with their respective crystallographic conformations is illustrated in Figure 3.

### 3.2. Virtual Screening (Targeting 3CLpro)

The virtual screening campaign identified 119 shogaol structural analogs that met the similarity threshold (Tanimoto coefficient ≥ 0.7). After docking these compounds against 3CLpro, several analogs exhibited significantly improved binding affinities compared to the parent compound (shogaol, −5.90 kcal/mol). Table 2 presents the top 10 shogaol-derived compounds ranked by their predicted binding affinities for 3CLpro.

The most promising compound, CHEMBL4472440, exhibited a binding affinity of −7.61 kcal/mol, representing a significant improvement of 1.71 kcal/mol over the parent compound shogaol, while maintaining a similarity score of 0.727. Similarly, CHEMBL218715 and CHEMBL423261 showed substantial enhancements in predicted binding, with affinities of −7.58 and −7.40 kcal/mol, respectively. The majority of the highest-ranked compounds demonstrated binding energies better than −6.90 kcal/mol, with improvements ranging from −1.05 to −1.71 kcal/mol compared to the parent compound, suggesting that alterations to the basic shogaol structure can substantially improve interactions with the 3CLpro-binding pocket. Interestingly, the molecule with the most powerful binding (CHEMBL4472440) did not possess the closest structural resemblance to shogaol; CHEMBL464274, despite having a considerably higher similarity index of 0.819, displayed strong binding (−7.14 kcal/mol) yet only placed fourth overall. This finding indicates that specific structural alterations, even those that decrease the overall resemblance to the original compound, may enhance interactions with the 3CLpro active site.

The similarity search for 6-gingerol identified a total of 399 compounds that met the similarity threshold criteria (Tanimoto coefficient ≥ 0.7). Of these, one compound failed during the SMILES-to-PDBQT conversion process, resulting in a final screening library of 398 6-gingerol analogs. Virtual screening of these 6-gingerol structural analogs against 3CLpro revealed numerous compounds with significantly improved binding affinities compared to the parent compound (6-gingerol, −5.90 kcal/mol). Table 3 presents the top 10 6-gingerol-derived compounds ranked by their predicted binding affinities.

The screening campaign for 6-gingerol analogs produced even more promising results than those observed for shogaol derivatives. The top-performing compound, CHEMBL1495225, exhibited a favorable binding affinity of −8.04 kcal/mol, representing an enhancement of 2.14 kcal/mol over the parent compound. This compound also maintained a relatively high similarity score of 0.766 to 6-gingerol, suggesting that its structural modifications preserved key pharmacophoric features while enhancing binding interactions. CHEMBL402213 and CHEMBL120931 also showed excellent binding profiles, with affinities of −7.86 and −7.54 kcal/mol, respectively.

Notably, the binding affinities of the top 10 compounds derived from 6-gingerol ranged from −7.26 to −8.04 kcal/mol, suggesting that the 6-gingerol scaffold may offer greater potential for optimization against 3CLpro compared to shogaol. CHEMBL1495225 displayed a binding affinity that was 0.43 kcal/mol stronger than that of the best shogaol analog (CHEMBL4472440, −7.61 kcal/mol), highlighting the superior inhibitory potential of this series.

The similarity search for S-allyl-cysteine identified only 18 compounds that met the similarity threshold criteria (Tanimoto coefficient ≥ 0.7), representing a substantially smaller analog pool compared to those generated for shogaol (119 compounds) and 6-gingerol (399 compounds). This limited structural diversity likely reflects the simpler chemical scaffold of S-allyl-cysteine and its relative scarcity in bioactive compound databases. The virtual screening of these S-allyl-cysteine analogs against 3CLpro revealed a different pattern compared to the shogaol and 6-gingerol series. Table 4 presents the S-allyl-cysteine-derived compounds ranked by their predicted binding affinities.

In contrast to the results observed for shogaol and 6-gingerol analogs, the structural modifications applied to the S-allyl-cysteine scaffold did not yield improvements in binding affinity against 3CLpro. The best-performing compound, CHEMBL2048655, exhibited a binding affinity of −5.05 kcal/mol, which is 0.45 kcal/mol weaker than that of the parent compound (S-allyl-cysteine, −5.50 kcal/mol). Similarly, all other analogs in this series showed diminished binding compared to S-allyl-cysteine.

Interestingly, CHEMBL2105861, which showed the highest structural similarity to S-allyl-cysteine (0.905), ranked third in binding affinity (−4.56 kcal/mol), suggesting that even minimal structural modifications to this scaffold may disrupt critical interactions with the 3CLpro-binding site. The compounds with moderate similarity scores (0.826), CHEMBL2048655 and CHEMBL464166, performed relatively better than those with lower similarity, but they still failed to match the binding affinity of the parent compound.

The reduced binding strength observed throughout the S-allyl-cysteine analog group implies that either the original compound’s structure is already ideal for 3CLpro interactions, or the specific modifications examined in our investigation disrupted essential binding elements. These analogs exhibited binding energies between −4.15 and −5.05 kcal/mol, considerably weaker than the leading compounds derived from shogaol (−7.61 kcal/mol) and 6-gingerol (−8.04 kcal/mol) frameworks.

### 3.3. Virtual Screening (Targeting PLpro)

The 119 shogaol analogs identified through the similarity search (Tanimoto coefficient ≥ 0.7) were also screened against PLpro, resulting in some of the highest-scoring compounds identified in this virtual screening campaign. Table 5 presents the top 10 shogaol-derived compounds ranked by their predicted binding affinities for PLpro.

The screening of shogaol analogs against PLpro yielded the highest-scoring compound identified in this entire virtual screening campaign. CHEMBL1720210 exhibited an exceptional binding affinity of −9.34 kcal/mol, representing a substantial improvement of 1.74 kcal/mol over the parent compound (shogaol, −7.60 kcal/mol). This binding affinity is significantly stronger than that of the best compounds identified from the 6-gingerol series (CHEMBL2177676, −8.83 kcal/mol) and the 8-gingerol series (CHEMBL4069090, −8.42 kcal/mol).

Multiple other shogaol analogs also demonstrated promising predicted affinity profiles, with nine compounds exhibiting binding affinities below −8.40 kcal/mol. CHEMBL1872436 and CHEMBL1513854 showed particularly strong binding, with affinities of −8.84 and −8.73 kcal/mol, respectively. Interestingly, CHEMBL1729115, which had the lowest similarity score to shogaol (0.702) among the top compounds, still exhibited a remarkably notable predicted affinity of −8.70 kcal/mol, indicating that more substantial structural deviations from the parent scaffold can still yield predicted PLpro binders.

Analysis of 398 structural variants related to 6-gingerol (previously identified during 3CLpro screening) against PLpro identified multiple compounds that displayed substantially improved binding energies compared to the original molecule. Within this collection of 399 similarity-derived compounds (one failed during SMILES-to-PDBQT conversion), many exhibited more favorable binding affinities with PLpro (ranging from −8.20 to −8.83 kcal/mol) than with 3CLpro. The ten most promising 6-gingerol derivatives, organized according to their predicted PLpro binding strengths, are presented in Table 6.

The screening results for 6-gingerol analogs against PLpro were particularly promising, with multiple compounds exhibiting binding affinities below −8.20 kcal/mol. The top-performing compound, CHEMBL2177676, demonstrated an exceptional binding affinity of −8.83 kcal/mol, representing a substantial improvement of 2.13 kcal/mol compared to the parent compound (6-gingerol, −6.70 kcal/mol). Interestingly, this compound had a relatively modest similarity score of 0.716, suggesting that certain structural modifications that deviate from the parent scaffold can improve binding affinity by up to 2.13 kcal/mol compared to the parent compound.

CHEMBL1231277 and CHEMBL4101529 also showed good binding profiles, with affinities of −8.78 and −8.71 kcal/mol, respectively, both maintaining a similarity score of 0.740 to the parent compound. The compound with the highest structural similarity to 6-gingerol in this set, CHEMBL1495225 (similarity score: 0.766), ranked fourth, with a binding affinity of −8.46 kcal/mol.

The binding affinities observed for 6-gingerol analogs against PLpro were generally higher than those observed for 3CLpro, suggesting that the 6-gingerol scaffold may be particularly suitable for targeting the PLpro-binding site. The compound with the highest affinity for PLpro (CHEMBL2177676, −8.83 kcal/mol) showed a binding affinity 0.79 kcal/mol stronger than that of the best 3CLpro inhibitor (CHEMBL1495225, −8.04 kcal/mol) from the same analog series.

After removing duplicate compounds that were already identified in the 6-gingerol similarity search, the virtual screening campaign for 8-gingerol identified 65 unique compounds that met the similarity threshold criteria (Tanimoto coefficient ≥ 0.7). This filtering of duplicates was essential to avoid redundancy, as the structural similarity between 6-gingerol and 8-gingerol resulted in considerable overlap in their respective analog libraries. The virtual screening of these unique 8-gingerol analogs against PLpro yielded several compounds with promising binding affinities. Table 7 presents the top 10 8-gingerol-derived compounds, ranked by their predicted binding affinities.

The top-performing 8-gingerol analog, CHEMBL4069090, exhibited a binding affinity of −8.42 kcal/mol, representing a significant improvement of 1.82 kcal/mol over the parent compound (8-gingerol, −6.60 kcal/mol). CHEMBL1448601 and CHEMBL1819482 also demonstrated favorable docking scores, with affinities of −8.30 and −8.03 kcal/mol, respectively. The binding affinities across the top 10 compounds ranged from −7.49 to −8.42 kcal/mol, with the similarity scores clustering narrowly between 0.725 and 0.754, indicating a relatively homogeneous group of structural analogs. Notably, while the 8-gingerol analogs showed substantial improvements over their parent compound, they were slightly less effective than the 6-gingerol series; the top 8-gingerol analog (CHEMBL4069090, −8.42 kcal/mol) exhibited a binding affinity 0.41 kcal/mol weaker than that of the best 6-gingerol analog (CHEMBL2177676, −8.83 kcal/mol). This differential performance between structurally similar parent compounds highlights the importance of exploring diverse structural starting points in the search for optimal protease inhibitors.

### 3.4. Binding Mode Analysis (Interactions with 3CLpro)

**Figure 4:**
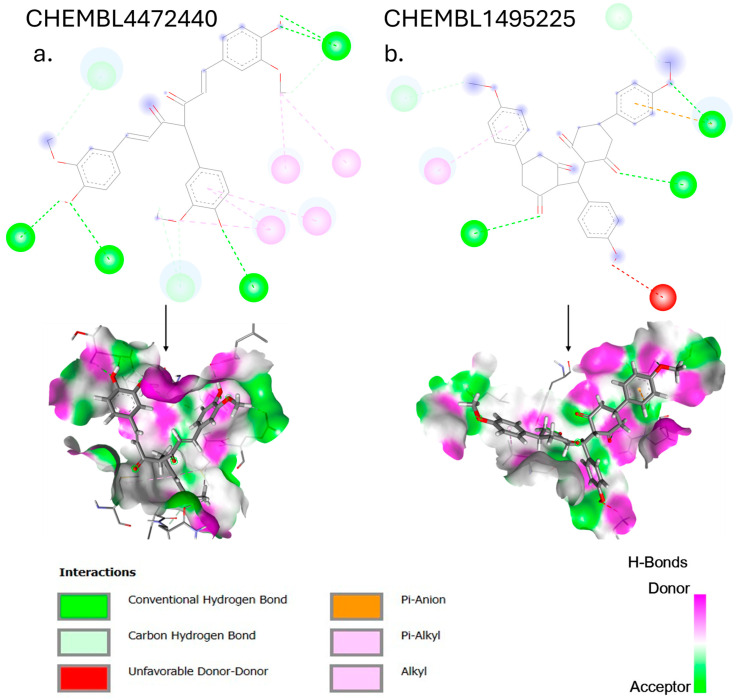
The 2D and 3D interaction diagrams showing the binding modes of top-scoring compounds with SARS-CoV-2 3CLpro: (**a**) CHEMBL4472440 (shogaol analog, binding affinity: −7.61 kcal/mol), (**b**) CHEMBL1495225 (6-gingerol analog, binding affinity: −8.04 kcal/mol. The 3D representations (bottom panels) show compounds within the PLpro-binding pocket, with surface coloring indicating hydrophobic and hydrophilic regions.

The compound with the highest affinity derived from shogaol, CHEMBL4472440, exhibits a binding affinity of −7.61 kcal/mol with 3CLpro. Analysis of the 2D interaction diagram (Figure 4) reveals an extensive network of binding interactions. The compound forms hydrogen bonds with several key residues, including HIS163 within the S1 subsite, THR26 at the binding pocket entrance, ARG188 (which helps anchor the ligand), and LEU141. Additionally, weaker polar interactions are observed with GLN189 and GLU166, the latter being important for substrate recognition.

The binding stability is further enhanced by hydrophobic interactions with CYS145 (a critical residue in the catalytic dyad), MET49 and MET165 (which form part of the hydrophobic S2 subsite), and LEU27. These multiple favorable interactions align with the observed binding affinity and explain the substantial improvement of 1.71 kcal/mol over the parent compound shogaol (−5.90 kcal/mol).

The structural modifications in CHEMBL4472440 appear strategically advantageous, particularly through the optimal positioning of hydroxyl groups that facilitate hydrogen bonding with key residues in the binding pocket. These structural adaptations allow the compound to effectively occupy multiple subsites of the 3CLpro-binding pocket, including the catalytically important regions, potentially interfering with the protease’s function. CHEMBL1495225, the top 6-gingerol analog, with a binding affinity of −8.04 kcal/mol, establishes multiple strong interactions within the 3CLpro-binding pocket. The compound forms several hydrogen bonds: ASP197, ARG131, TYR239, LEU272, and GLY195. There is only one hydrophobic bond with LEU287, but it forms an unfavorable bond with LYS5. This binding pattern suggests that the structural features of CHEMBL1495225, derived from the 6-gingerol scaffold, are particularly well suited for 3CLpro inhibition, explaining the substantial improvement of 2.14 kcal/mol over the parent compound (6-gingerol, −5.90 kcal/mol).

### 3.5. Binding Mode Analysis (Interactions with PLpro)

**Figure 5:**
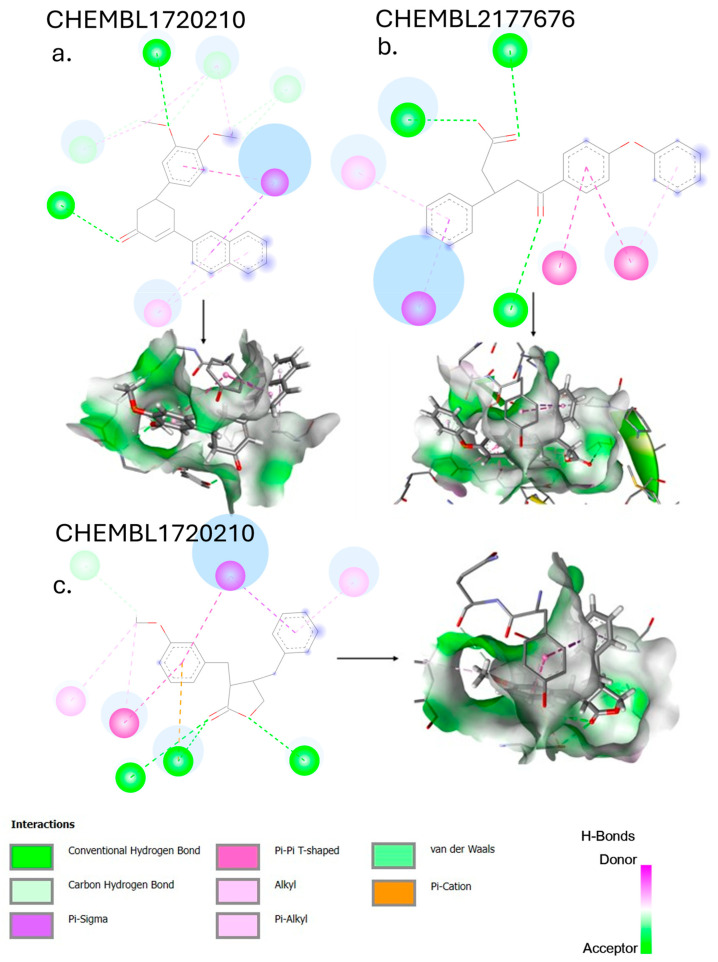
The 2D and 3D interaction diagrams illustrating the binding modes of top-scoring compounds with SARS-CoV-2 PLpro: (**a**) CHEMBL1720210 (shogaol analog, binding affinity: −9.34 kcal/mol), (**b**) CHEMBL2177676 (6-gingerol analog, binding affinity: −8.83 kcal/mol), and (**c**) CHEMBL4069090 (8-gingerol analog, binding affinity: −8.42 kcal/mol). The 3D representations (bottom panels) show compounds within the PLpro-binding pocket, with surface coloring indicating hydrophobic) and hydrophilic regions.

CHEMBL1720210, the compound with the highest binding affinity (−9.34 kcal/mol) in the entire virtual screening campaign, establishes an extensive network of interactions with the PLpro-binding pocket. Figure 5 depicts several key binding features: hydrogen bonds with GLY163, LEU162, GLN269, TYR265, and TYR273, and hydrophobic interactions with TYR268 and PRO248. The exceptional binding affinity of CHEMBL1720210 (−9.34 kcal/mol) can be attributed to this combination of hydrogen bonds and hydrophobic interactions that collectively provide optimal positioning within the PLpro-binding site. The compound appears to exploit key interactions with the binding pocket residues more effectively than any other screened compound.

CHEMBL2177676, the top 6-gingerol analog, has a binding affinity of −8.83 kcal/mol against PLpro. The ligand–protein complex is maintained by the following bonds: THR301, ASP164, PRO248, TYR268, TYR273, GLY163, and LEU162. Compared to the parent compound 6-gingerol (−6.70 kcal/mol), CHEMBL2177676 shows a substantial improvement of 2.13 kcal/mol in binding affinity. The structural modifications appear to have enhanced the compound’s ability to form multiple types of interactions.

Based on Figure 5, CHEMBL4069090 was the top 8-gingerol analog, with a binding affinity of −8.42 kcal/mol against PLpro.

This compound interacts with ASP164, THR301, TYR273, TYR268, TYR264, PRO248, LEU162, and GLN269. The binding mode of CHEMBL4069090 shows interesting similarities to CHEMBL2177676 (the top 6-gingerol analog), particularly in its interactions with ASP164 and THR301 through the carboxyl/ester group. However, CHEMBL4069090 appears to form a more extensive network of aromatic interactions with TYR264 and TYR268, suggesting a slightly different orientation that may account for the difference in binding affinity (−8.42 kcal/mol versus −8.83 kcal/mol for CHEMBL2177676).

The structural comparison between the parent compounds and their top-performing analogs reveals key modifications that enhance protease binding. The transformation of shogaol’s linear α,β-unsaturated ketone system into CHEMBL1720210’s rigid cyclohexanone core, connected to a 2-naphthyl substituent via an α,β-unsaturated system, introduces an extended aromatic system that enables additional π-π stacking interactions with PLpro’s TYR268 and TYR273 residues, while replacing the hydroxyl group with a methoxy group and maintaining the dimethoxyphenyl group, collectively accounting for the improvement in binding affinity. The transformation of 6-gingerol’s linear aliphatic chain with a single ketone into CHEMBL1495225’s dual cyclohexane-1,3-dione system, bridged by a carbon bearing a para-hydroxyphenyl group and decorated with two additional para-methoxyphenyl substituents, creates a rigid, three-dimensional scaffold that positions multiple hydrogen bond acceptors (from the four ketone groups) and three aromatic rings for optimal interactions with 3CLpro; this architectural change from a flexible linear chain to a rigid multi-ring system with strategically placed aromatic groups enables interactions with multiple binding site residues, including ASP197, ARG131, TYR239, LEU272, and GLY195, accounting for the improvement in binding affinity. The transformation of 8-gingerol’s linear chain, containing a ketone and hydroxyl group, into CHEMBL4069090’s compact γ-butyrolactone core, with benzyl and meta-methoxybenzyl substituents, dramatically reduces its conformational flexibility, while the replacement of the hydrogen bond donor groups (two OH groups in 8-gingerol) with a lactone ester enhances the membrane permeability and BBB penetration, and the two aromatic rings provide hydrophobic interactions with PLpro residues, collectively accounting for the improvement in binding affinity and superior ADMET profile.

### 3.6. ADMET Properties of the Top-Scoring Compounds

SwissADME predicts a wide range of physicochemical characteristics, pharmacokinetic parameters, drug-like properties, and medicinal chemistry friendliness. For characteristics including lipophilicity, water solubility, gastrointestinal absorption, blood–brain barrier permeability, and interactions with cytochrome P450 enzymes, the tool employs verified predictive models. Furthermore, adherence to recognized drug-likeness guidelines is assessed, including Lipinski’s Rule of Five [37].

For 3CLpro: CHEMBL4472440 (shogaol analog), CHEMBL1495225 (6-gingerol analog); for PLpro: CHEMBL1720210 (shogaol analog), CHEMBL2177676 (6-gingerol analog), and CHEMBL4069090 (8-gingerol analog). Table 8 presents a comprehensive comparison of their physicochemical properties, predicted pharmacokinetic parameters, and drug-likeness metrics.

The compounds’ molecular weights vary from 540.60 g/mol (CHEMBL1495225) to 296.36 g/mol (CHEMBL4069090). The shogaol-derived CHEMBL4472440 (504.53 g/mol) and 6-gingerol-derived CHEMBL1495225 (540.60 g/mol) both slightly surpass the optimal range recommended by Lipinski’s Rule of Five (<500 g/mol), which may affect their oral bioavailability. However, CHEMBL4069090, CHEMBL1720210, and CHEMBL2177676 are all within this range [38].

The number of rotatable bonds in the compounds indicates their differing levels of flexibility. While CHEMBL1720210 exhibits the lowest conformational flexibility (4 rotatable bonds), indicating a more rigid structure that might be responsible for its binding affinity for PLpro, CHEMBL4472440 exhibits the highest conformational flexibility (11 rotatable bonds), surpassing Veber’s criterion for optimal oral bioavailability (≤10 rotatable bonds). The topological polar surface area (TPSA) values reveal marked differences in polarity. CHEMBL1720210 and CHEMBL4069090 both have significantly lower TPSA values (35.53 Å^2^) compared to CHEMBL4472440 (122.52 Å^2^), explaining their predicted ability to cross the blood–brain barrier while the others cannot [39].

The hydrogen bonding capacity differs significantly among the compounds. CHEMBL4472440 has the highest number of hydrogen bond acceptors (eight) and donors (three), while CHEMBL1720210 has no hydrogen bond donors and only three acceptors. This variation explains their different interaction patterns with the protease-binding sites, with CHEMBL4472440 forming multiple hydrogen bonds with 3CLpro residues and CHEMBL1720210 relying more on hydrophobic and π-π interactions with PLpro.

All of the compounds are very lipophilic, which is consistent with their capacity to interact with the hydrophobic binding pockets of the proteases, according to the consensus LogP values, which vary from 3.55 to 4.55 (the average of all of the methods employed by Swiss to determine LogP). In accordance with the extensive hydrophobic interactions found in the investigation of its binding mechanism, CHEMBL1720210 exhibits the highest degree of lipophilicity (consensus LogP: 4.55) [40,41,42].

Aqueous solubility predictions (ESOL LogS, Ali LogS, and Silicos-IT LogSw) consistently classify these compounds as moderately to poorly soluble. CHEMBL4069090 demonstrates the most favorable solubility profile across all three prediction methods, while CHEMBL1495225 shows the poorest solubility according to Silicos-IT LogSw (−8.90) [43,44].

All five compounds are predicted to have high gastrointestinal absorption, suggesting good oral bioavailability despite their varying physicochemical properties. Interestingly, only CHEMBL1720210 and CHEMBL4069090 are predicted to cross the blood–brain barrier. The drugs interact differently with a key efflux transporter, P-gp. It is anticipated that CHEMBL1495225 and CHEMBL2177676 are P-gp substrates, which may restrict their tissue distribution and bioavailability.

Significant variations in the possible dangers of drug–drug interactions are shown by the cytochrome P450 (CYP) inhibition profiles. While CHEMBL2177676 inhibits four of the five major CYP isoforms (CYP2C19, CYP2C9, CYP2D6, and CYP3A4), indicating a higher likelihood of drug–drug interactions, CHEMBL4069090 has the most favorable profile, inhibiting just CYP2C19 and CYP2C9. The inhibition of CYP1A2, CYP2C19, CYP2C9, and CYP3A4 by CHEMBL1720210 may be troublesome, because CYP3A4 is involved in the metabolism of around half of the medications that are marketed [45].

It is evident that while both CHEMBL4472440 and CHEMBL1495225 fail to meet one of the criteria (namely, molecular weight), the remaining compounds (namely, CHEMBL1720210, CHEMBL2177676, and CHEMBL4069090) demonstrate compliance with all five of Lipinski’s conditions. In contrast to the other chemicals, CHEMBL2177676 exhibits the greatest bioavailability score (0.85), which may indicate greater oral bioavailability.

These chemicals are unlikely to result in false positives in biological tests, because none of them cause pan-assay interference chemicals (PAINS) alarms. However, from the standpoint of drug development, CHEMBL4472440 and CHEMBL1495225 show Brenk alerts (two and one, respectively), which may suggest the presence of hazardous fragments [46,47]. The synthetic accessibility scores range from 2.87 (CHEMBL2177676) to 4.28 (CHEMBL1495225), with lower values indicating easier synthesis. It is evident that CHEMBL2177676 and CHEMBL4069090 (3.02) appear to be the most synthetically accessible, which may facilitate their further development and optimization.

Obviously, the combination of favorable binding affinity (−9.34 kcal/mol) with beneficial drug-like characteristics, including BBB permeability and the absence of P-gp substrate liability, renders CHEMBL1720210 a notably promising candidate for PLpro inhibition. The primary limitation of this approach is the inhibition of multiple cytochrome P450 (CYP) enzymes, which has the potential to result in drug–drug interactions.

With regard to 3CLpro inhibition, CHEMBL1495225 demonstrates the highest binding affinity (−8.04 kcal/mol); however, it presents several ADMET challenges, including high molecular weight, poor predicted solubility, and P-gp substrate liability. CHEMBL4472440, which exhibits a marginally diminished binding affinity (−7.61 kcal/mol), provides a more balanced ADMET profile, notwithstanding its failure to attain the optimal molecular weight threshold. CHEMBL4069090 exhibits a potentially optimal overall profile, demonstrating robust binding affinity for PLpro (−8.42 kcal/mol) and commendable drug-like characteristics, including the most compact molecular structure, favorable synthetic accessibility, a conducive CYP inhibition profile, and BBB permeability. This balanced profile renders it a particular attractive candidate for further development.

### 3.7. Analysis of Dual-Targeting Compounds

The identification of compounds with dual-targeting capabilities against both 3CLpro and PLpro represents a particularly promising outcome of this virtual screening campaign. Among the 6-gingerol analogs, CHEMBL1495225 demonstrated dual-binding properties, with affinities of −8.04 kcal/mol for 3CLpro and −8.46 kcal/mol for PLpro, representing improvements of 2.14 and 1.76 kcal/mol over the parent compound, respectively. CHEMBL4101529, also derived from the 6-gingerol scaffold, exhibited favorable predicted binding to both proteases (3CLpro: −7.36 kcal/mol; PLpro: −8.71 kcal/mol), while CHEMBL4083532 showed comparable dual activity (3CLpro: −7.27 kcal/mol; PLpro: −8.42 kcal/mol). From the shogaol series, CHEMBL1514823 (3CLpro: −6.97 kcal/mol; PLpro: −8.63 kcal/mol) and CHEMBL1355942 (3CLpro: −6.95 kcal/mol; PLpro: −8.44 kcal/mol) also demonstrated dual-binding capabilities, albeit with stronger affinity for PLpro. The ability of these compounds to effectively target both viral proteases suggests their potential as multi-target inhibitors, which could significantly reduce the likelihood of viral resistance development through mutations in a single target protein. This dual-targeting approach has proven successful in the treatment of HIV, where combination therapies or single agents targeting multiple viral proteins have become the standard approach [48].

The structural analysis of these dual-binding compounds reveals interesting patterns across the different scaffolds. The 6-gingerol-derived compounds (CHEMBL1495225, CHEMBL4101529, and CHEMBL4083532) showed the most balanced binding profiles, with strong affinities for both proteases, suggesting that the 6-gingerol scaffold provides an optimal template for dual inhibition. In contrast, the shogaol-derived dual inhibitors (CHEMBL1514823 and CHEMBL1355942) exhibited a preference for PLpro over 3CLpro, with binding affinity differences exceeding 1.5 kcal/mol. These structural elements appear to confer the flexibility and complementary functionality necessary to accommodate the distinct binding-site architectures of both proteases. While 3CLpro features a catalytic dyad (His41-Cys145) and PLpro contains a catalytic triad (Cys111-His272-Asp286), both active sites contain hydrophobic pockets and hydrogen bond donors/acceptors that can be exploited by compounds with appropriate structural versatility [29,32]. Among the compounds with complete ADMET analysis, CHEMBL1495225 demonstrated dual-targeting capability, although its high molecular weight (540.60 g/mol) and P-gp substrate liability may present development challenges. Future studies employing molecular dynamics simulations would be valuable to confirm the stability of these dual-binding modes and to guide structural optimization efforts aimed at enhancing potency against both targets while maintaining favorable pharmacological properties.

### 3.8. Toxicity Risk Assessment Using ProTox-III

ProTox-III is an online tool that predicts the toxicity of small chemicals in silico. For predicting a variety of toxicological endpoints, ProTox-III employs a comprehensive approach to toxicity prediction that combines machine learning algorithms, fragment-based techniques, and chemical similarity. There are two qualitative predictions for each compound generated by ProTox-III: an active or inactive prediction, and a probability score (ranging from 0 to 1), with a higher probability score indicating greater confidence in the prediction. The highest degree of confidence in predictions is indicated by values near 1.0, with values above 0.5 being considered more reliable [35].

This study evaluated the toxicity profiles of five promising compounds (CHEMBL4472440, CHEMBL1495225, CHEMBL1720210, CHEMBL2177676, and CHEMBL4069090) identified through virtual screening. The researchers used ProTox-III to analyze the SMILES chemical notations of these compounds, focusing particularly on potential organ toxicity and specific toxicity endpoints that could affect their development as therapeutic agents. Toxicology is a critical component of drug safety assessment, as adverse effects of a serious nature or the discontinuation of treatment can be caused by compounds that damage vital organs. Table 9 presents the organ toxicity predictions for the five lead compounds identified in our virtual screening campaign.

Each lead compound exhibits distinct patterns of organ toxicity. The analysis revealed that four compounds (CHEMBL4472440, CHEMBL1495225, CHEMBL1720210, and CHEMBL4069090) are unlikely to cause liver damage, showing inactive hepatotoxicity profiles with relatively high confidence scores, ranging from 0.60 to 0.70. However, CHEMBL2177676 showed potential liver toxicity concerns, albeit with a borderline probability score of 0.50. CHEMBL1495225 and CHEMBL2177676 had the highest odds of being inactive, according to the neurotoxicity predictions (0.75 and 0.89, respectively), indicating a lower chance of negative neurological consequences. CHEMBL1720210 has a comparatively low confidence score (0.54), indicating that it may be neurotoxic. The results of the nephrotoxicity assessment indicate potential renal concerns for CHEMBL4472440, CHEMBL1495225, CHEMBL4069090, and CHEMBL2177676, with the latter demonstrating the highest probability score (0.70). This finding suggests that kidney function monitoring may be indicated for compounds that progress to preclinical development. Predictions of respiratory and cardiac toxicity generally indicate that all of the compounds are inactive. Table 10 presents the toxicity endpoint predictions for the five lead compounds.

In addition to organ-specific toxicity, various toxicity endpoints were assessed to provide a comprehensive toxicological profile of the lead compounds. These endpoints encompass critical safety parameters, including carcinogenicity, mutagenicity, immunotoxicity, and other specialized toxicity concerns. All five compounds were predicted to be non-carcinogenic, with CHEMBL2177676 demonstrating the highest confidence score (0.79). This favorable prediction regarding carcinogenicity is particularly significant for therapeutic development, as carcinogenic potential represents a critical safety concern in the evaluation of drug candidates.

The immunotoxicity predictions exhibited considerable variation among the compounds. CHEMBL4472440, CHEMBL1720210, and CHEMBL4069090 exhibited potential immunotoxic effects, with high confidence scores (0.95, 0.62, and 0.95, respectively). In contrast, CHEMBL1495225 and CHEMBL2177676 were predicted to be non-immunotoxic, with high confidence scores (0.98 and 0.99, respectively). Regarding mutagenicity, all of the compounds were predicted to be non-mutagenic, with CHEMBL2177676 exhibiting the highest confidence score (0.89) for the absence of mutagenic potential. This favorable profile mitigates concerns regarding genotoxicity and potential long-term genetic damage. With confidence values ranging from 0.72 to 0.88, all of the substances showed a modest potential for cytotoxicity. The most favorable cytotoxicity profile (0.88) was shown by CHEMBL2177676, suggesting a lower risk of overall cellular damage.

With comparatively high confidence scores (0.68–0.82), all of the drugs were expected to show possible toxicity associated with the blood–brain barrier. The SwissADME investigation, which showed that CHEMBL1720210 and CHEMBL4069090 can cross the blood–brain barrier, is in line with this prediction. This finding needs to be carefully considered, especially in the light of any possible negative neurological repercussions. The ecotoxicity assessment revealed that CHEMBL4472440, CHEMBL1720210, and CHEMBL4069090 present potential environmental toxicity concerns, while CHEMBL1495225 and CHEMBL2177676 were predicted to have reduced environmental impact. The clinical toxicity predictions indicated that CHEMBL1495225 and CHEMBL2177676 may exhibit potential adverse effects in clinical settings (confidence scores of 0.61 and 0.58, respectively). The remaining compounds demonstrated more favorable profiles for this endpoint. All of the compounds were predicted to be inactive for nutritional toxicity, suggesting minimal interference with nutritional pathways or metabolic processes.

### 3.9. Drug-Induced Gene Expression Prediction

The compounds acknowledged as possessing the greatest potential for interaction with the target proteases were subsequently evaluated based on mRNA-based predictive outcomes to determine their capacity for forecasting drug-induced alterations in gene expression profiles related to SARS-CoV-2. These mRNA-based prediction results involve estimates generated from the examination of gene expression at the mRNA level. These predictions are crucial for assessing the impact of a drug or substance on gene transcription and subsequent regulation of cellular activities.

DIGEP-Pred is an in silico tool that predicts changes in gene expression (upregulation or downregulation) induced by a compound on human genes. Pa (i.e., probability “active”) represents the likelihood that the compound will alter the expression of a specific gene (up or down). The higher the Pa, the more likely the effect is to be real. Genes with relevance to SARS-CoV-2, particularly those involved in inflammation, antioxidation, and other relevant pathways, were selected and evaluated based on these predictions [36]. Table 11 presents the DIGEP-Pred results for the drug-induced changes in the gene expression profiles of the compounds tested. The table summarizes the key genes that were predicted to be upregulated (↑) or downregulated (↓), along with their corresponding probability values (Pa), which indicate the likelihood of these changes occurring.

CHEMBL4472440 is predicted to downregulate *CXCL2* and *TNFRSF10B*, suggesting anti-inflammatory and anti-apoptotic effects [49]. Simultaneously, it upregulates key antioxidant genes such as *GCLM*, *GCLC*, *NQO1*, and *SOD3*, indicating a strong potential to reduce oxidative stress [50]. These combined effects support its adjuvant therapeutic potential in COVID-19.

CHEMBL1495225 shows downregulation of *CCL5*, indicating anti-inflammatory potential [51]. It upregulates *GAS6*, with possible antioxidant and tissue repair roles, although the increase in several cell-cycle regulators suggests a need for cautious interpretation regarding apoptosis [52].

CHEMBL1720210 shows downregulation of *CXCL2* and *PLCG2*, suggesting anti-inflammatory properties [49]. The upregulation *GAS6* points to antioxidant and regenerative potential. The increase in cell cycle-related genes (i.e., *AURKA*) suggests caution, as they might enhance cell division but could also aid in tissue repair [52].

CHEMBL4069090 shows an increase in *ENC1*, which is beneficial for cellular protection and oxidative stress modulation. The upregulation of *CASP2* may indicate a pro-apoptotic effect [53]. However, since the Pa values for the genes potentially regulated by the tested compound are all below 0.7, the predictions are less certain, suggesting that the observed effects may not be as strong or reliable as those with higher Pa values.

Several methodological limitations must be acknowledged when interpreting the results of this preliminary investigation. The current study relies primarily on molecular docking and scoring functions to predict binding affinities, without implementing more advanced computational techniques, such as molecular dynamics simulations, which would provide information about the stability of protein–ligand complexes over time and would account for protein flexibility. Similarly, no free binding energy calculations were performed that could provide more accurate estimates of binding strength. These computational limitations, although common in initial virtual screening campaigns, represent important considerations for the future refinement of these promising compounds.

The scope of our compound selection represents another limitation of this study. By focusing exclusively on structural analogs derived from two plant species (*Zingiber officinale* and *Allium sativum* L.), we may have overlooked novel scaffolds from other natural sources with potential anti-SARS-CoV-2 activity. Our reliance on the ChEMBL database, while providing experimentally validated compounds, restricts the chemical space explored compared to theoretical compound libraries. Additionally, our similarity threshold criterion (Tanimoto coefficient ≥ 0.7) was chosen to ensure structural relevance to the parent compounds but may have excluded more structurally diverse molecules with potential activity. Future studies should consider expanding to include other medicinal plants with documented antiviral properties and broader natural product databases, such as ZINC.

The absence of experimental validation constitutes another significant limitation of this work. In vitro enzyme inhibition tests would be necessary to confirm the actual inhibitory potency of the identified compounds against 3CLpro and PLpro. Additionally, cellular antiviral tests would be needed to demonstrate efficacy in reducing viral replication, while cytotoxicity studies would help validate the safety profiles predicted by our in silico approaches. Gene expression predictions, although informative, also require experimental confirmation through techniques such as reverse-transcription polymerase chain reaction or RNA-Seq analysis to verify the effects of compounds on inflammatory and antioxidant pathways. This lack of experimental support substantially limits the interpretive power of our findings, as the predicted interactions may not translate into actual biological activity.

The computational prediction tools used for ADMET and toxicity assessment have inherent limitations, including defined applicability domains and varying prediction accuracies across different chemical spaces. The clinical translation of our findings must also consider the complex pharmacokinetics of natural products, potential drug–drug interactions in COVID-19 treatment regimens, and the emergence of viral variants that may affect protease structures and inhibitor efficacy.

Despite these limitations, the systematic computational approach used in this study provides a solid foundation for subsequent investigations. The comprehensive workflow, combining similarity-based analog generation, extensive virtual screening, detailed binding mode analysis, and rigorous ADMET profiling and mRNA-based gene expression prediction, successfully establishes a structured framework for prioritizing compounds for future experimental studies. Rather than diminishing the value of the current findings, these limitations represent natural progression points in the drug discovery pipeline, as initial computational screening is an essential and resource-efficient first step in identifying promising compounds that merit further investigation through more intensive computational and experimental methodologies.

## 4. Conclusions

The present in silico study successfully identified several promising lead compounds with enhanced binding affinities to SARS-CoV-2 proteases compared to their natural product parent compounds from *Zingiber officinale* and *Allium sativum*. By combining similarity searches, molecular docking, binding mode analysis, and ADMET profiling, a multi-stage drug discovery method was developed to identify potential therapeutic candidates against critical viral targets.

The most significant findings from the screening process were CHEMBL1720210, a shogaol derivative demonstrating superior predicted binding affinity to PLpro (−9.34 kcal/mol, representing a 1.74 kcal/mol enhancement), and CHEMBL1495225, a 6-gingerol analog exhibiting promising predicted binding to 3CLpro (−8.04 kcal/mol, representing a 2.14 kcal/mol improvement). Notably, compounds such as CHEMBL1495225 and CHEMBL4101529 demonstrated dual-targeting capabilities against both proteases, suggesting their potential as multi-target inhibitors that could potentially reduce the development of viral resistance.

The binding mode analyses revealed that these compounds establish extensive networks of interactions with key residues in the protease-binding pockets. CHEMBL4472440 forms multiple hydrogen bonds with 3CLpro residues (HIS163, THR26, ARG188, LEU141), complemented by hydrophobic interactions with catalytically important residues, while CHEMBL1720210’s high PLpro binding can be attributed to optimal hydrogen bonding combined with strategic hydrophobic interactions. Interestingly, compounds with moderate structural similarity to their parent molecules often demonstrated superior binding compared to those with higher similarity scores, suggesting that certain strategic modifications can significantly enhance protease binding even when reducing the overall similarity to the parent compound.

The ADMET profiling revealed that CHEMBL4069090 exhibited the most favorable overall profile, combining strong PLpro-binding affinity (−8.42 kcal/mol) with advantageous drug-like properties, including a small molecular structure, good synthetic accessibility, and a favorable CYP inhibition profile. Despite CHEMBL1720210’s exceptional binding affinity, its inhibition of multiple CYP enzymes raised concerns about potential drug–drug interactions. Similarly, while CHEMBL1495225 demonstrated superior 3CLpro-binding affinity, it was hindered by high molecular weight and predicted poor solubility, among other ADMET limitations. Toxicity studies indicated that although all of the compounds were predicted to be non-mutagenic and non-carcinogenic, most exhibited specific concerns, particularly regarding nephrotoxicity and immunotoxicity.

Based on the DIGEP-Pred analysis of the five compounds, their potential as adjuvants in SARS-CoV-2 therapy seems promising. CHEMBL4472440 and CHEMBL1720210 showed upregulation of genes related to antioxidant and anti-inflammatory pathways (e.g., GCLM, SOD3), which could help modulate the immune response and reduce inflammation in SARS-CoV-2. However, further studies are needed to validate these in silico findings through in vitro and in vivo experiments to confirm their efficacy and safety as adjuvants in SARS-CoV-2 management.

## Figures and Tables

**Table 1:** Computational tools and resources used in this study.

Tool	Function
AutoDock Vina	Molecular docking
Protein Data Bank	Source 3CLpro and PLpro
SwissADME	ADME prediction
Swiss Similarity	Used for similarity searches
Open Babel	Used alongside RDKit for conversion
ChEMBL	Source of bioactive compounds for similarity searches
Python	Programming language
RDKit	Initial 3D structure generation
Custom Python Scripts	Conversion to PDBQT format
ProTox-III	Prediction of toxicity profiles
Discovery Studio Visualizer	Ligand–protein interaction analysis
DIGEP-Pred	mRNA-based prediction results

**Table 2:** Top 10 shogaol-derived compounds with highest binding affinities for 3CLpro.

Rank	Compound ID	Binding Affinity (kcal/mol)	Improvement over Shogaol (kcal/mol)	Similarity Score
1	CHEMBL4472440	−7.61	−1.71	0.727
2	CHEMBL218715	−7.58	−1.68	0.716
3	CHEMBL423261	−7.40	−1.50	0.709
4	CHEMBL464274	−7.14	−1.24	0.819
5	CHEMBL1720210	−7.19	−1.29	0.731
6	CHEMBL1591897	−7.06	−1.16	0.708
7	CHEMBL3828225	−6.97	−1.07	0.700
8	CHEMBL1514823	−6.97	−1.07	0.711
9	CHEMBL2397175	−6.95	−1.05	0.771
10	CHEMBL1355942	−6.95	−1.05	0.750

**Table 3:** Top 10 6-gingerol-derived compounds with highest binding affinities for 3CLpro.

Rank	Compound ID	Binding Affinity (kcal/mol)	Improvement over Shogaol (kcal/mol)	Similarity Score
1	CHEMBL1495225	−8.04	−2.14	0.766
2	CHEMBL402213	−7.86	−1.96	0.718
3	CHEMBL120931	−7.54	−1.64	0.758
4	CHEMBL1627776	−7.36	−1.46	0.718
5	CHEMBL4101529	−7.36	−1.46	0.740
6	CHEMBL4452832	−7.40	−1.50	0.769
7	CHEMBL1911177	−7.28	−1.38	0.743
8	CHEMBL4465547	−7.27	−1.37	0.750
9	CHEMBL4083532	−7.27	−1.37	0.740
10	CHEMBL1627554	−7.26	−1.36	0.718

**Table 4:** Top S-allyl-cysteine-derived compounds with highest binding affinities for 3CLpro.

Rank	Compound ID	Binding Affinity (kcal/mol)	Improvement over Shogaol (kcal/mol)	Similarity Score
1	CHEMBL2048655	−5.05	+0.45	0.826
2	CHEMBL464166	−4.84	+0.66	0.826
3	CHEMBL2105861	−4.56	+0.94	0.905
4	CHEMBL1555183	−4.52	+0.98	0.775
5	CHEMBL2010208	−4.24	+1.26	0.775
6	CHEMBL1967876	−4.27	+1.23	0.775
7	CHEMBL4540850	−4.28	+1.22	0.750
8	CHEMBL4077223	−4.35	+1.15	0.738
9	CHEMBL4083417	−4.41	+1.09	0.775
10	CHEMBL1741968	−4.15	+1.35	0.816

**Table 5:** Top 10 shogaol-derived compounds with highest binding affinities for PLpro.

Rank	Compound ID	Binding Affinity (kcal/mol)	Improvement over Shogaol (kcal/mol)	Similarity Score
1	CHEMBL1720210	−9.34	−1.74	0.731
2	CHEMBL1872436	−8.84	−1.24	0.750
3	CHEMBL1513854	−8.73	−1.13	0.716
4	CHEMBL1729115	−8.70	−1.10	0.702
5	CHEMBL1489722	−8.68	−1.08	0.731
6	CHEMBL1514823	−8.63	−1.03	0.711
7	CHEMBL1705415	−8.60	−1.00	0.716
8	CHEMBL1355942	−8.44	−0.84	0.750
9	CHEMBL1727607	−8.41	−0.81	0.731
10	CHEMBL2204311	−8.36	−0.76	0.765

**Table 6:** Leading compounds derived from 6-gingerol with the highest PLpro affinity.

Rank	Compound ID	Binding Affinity (kcal/mol)	Improvement over Shogaol (kcal/mol)	Similarity Score
1	CHEMBL2177676	−8.83	−2.13	0.716
2	CHEMBL1231277	−8.78	−2.08	0.740
3	CHEMBL4101529	−8.71	−2.01	0.740
4	CHEMBL1495225	−8.46	−1.76	0.766
5	CHEMBL4083532	−8.42	−1.72	0.740
6	CHEMBL2004966	−8.39	−1.69	0.742
7	CHEMBL4093334	−8.30	−1.60	0.747
8	CHEMBL3289509	−8.28	−1.58	0.733
9	CHEMBL1939193	−8.26	−1.56	0.732
10	CHEMBL1507403	−8.29	−1.59	0.731

**Table 7:** Leading 8-gingerol-based compounds showing highest affinity to PLpro.

Rank	Compound ID	Binding Affinity (kcal/mol)	Improvement over 8-Gingerol (kcal/mol)	Similarity Score
1	CHEMBL4069090	−8.42	−1.82	0.730
2	CHEMBL1448601	−8.30	−1.70	0.727
3	CHEMBL1819482	−8.03	−1.43	0.726
4	CHEMBL2324432	−8.02	−1.42	0.725
5	CHEMBL405043	−7.90	−1.30	0.729
6	CHEMBL50536	−7.60	−1.00	0.729
7	CHEMBL3322946	−7.59	−0.99	0.731
8	CHEMBL2393534	−7.56	−0.96	0.725
9	CHEMBL1939199	−7.49	−0.89	0.754
10	CHEMBL2324434	−7.49	−0.89	0.725

**Table 8:** Physicochemical properties and ADMET profiles of top-performing compounds against SARS-CoV-2 proteases.

Molecule	CHEMBL4472440	CHEMBL1495225	CHEMBL1720210	CHEMBL2177676	CHEMBL4069090
Formula	C_29_H_28_O_8_	C_33_H_32_O_7_	C_24_H_22_O_3_	C_23_H_20_O_4_	C_19_H_20_O_3_
Molecular Weight (g/mol)	504.53	540.6	358.43	360.4	296.36
Rotatable Bond Count	11	7	4	8	5
Hydrogen Bond Acceptors	8	7	3	4	3
Hydrogen Bond Donors	3	1	0	1	0
Topological Polar Surface Area (Å^2^)	122.52	106.97	35.53	63.6	35.53
Lipophilicity (iLogP)	3.23	3.42	3.5	2.77	2.74
Lipophilicity (XLogP3)	4.81	3.92	4.66	4.31	4
Lipophilicity (WLogP)	4.34	5.16	5.39	5.31	3.27
Lipophilicity (MLogP)	1.86	2.21	3.65	3.75	3.36
Lipophilicity (Silicos-IT)	5.44	6.05	5.55	4.81	4.37
Consensus Lipophilicity (LogP)	3.94	4.15	4.55	4.19	3.55
Water Solubility (ESOL LogS)	−5.63	−5.53	−5.17	−4.76	−4.27
Water Solubility (Ali LogS)	−7.12	−5.87	−5.13	−5.36	−4.45
Water Solubility (Silicos-IT LogS)	−6.44	−8.9	−7.88	−7.3	−6.12
Gastrointestinal Absorption	High	High	High	High	High
Blood–Brain Barrier Permeability	No	No	Yes	No	Yes
P-Glycoprotein Substrate	No	Yes	No	Yes	No
CYP1A2 Inhibition	No	No	Yes	No	No
CYP2C19 Inhibition	No	Yes	Yes	Yes	Yes
CYP2C9 Inhibition	Yes	No	Yes	Yes	Yes
CYP2D6 Inhibition	No	No	No	Yes	Yes
CYP3A4 Inhibition	Yes	Yes	Yes	Yes	No
Lipinski Rule Violations	1	1	0	0	0
Oral Bioavailability Score	0.55	0.55	0.55	0.85	0.55
PAINS Alert Count	0	0	0	0	0
Brenk Toxicophoric Count	2	1	0	0	0
Lead Likeness Violations	3	2	2	3	1
Synthetic Accessibility Score	4.17	4.28	3.65	2.87	3.02

PAINS, pan-assay interference chemicals.

**Table 9:** Organ toxicity predictions for lead compounds against SARS-CoV-2.

Compound	Organ Toxicity				
	Hepatotoxicity	Neurotoxicity	Nephrotoxicity	Respiratory	Cardiotoxicity
CHEMBL4472440	I/0.70	I/0.61	A/0.55	I/0.70	I/0.58
CHEMBL1495225	I/0.68	I/0.75	A/0.63	I/0.69	I/0.57
CHEMBL1720210	I/0.60	A/0.54	I/0.55	I/0.52	I/0.76
CHEMBL2177676	A/0.50	I/0.89	A/0.70	I/0.61	I/0.66
CHEMBL4069090	I/0.70	I/0.61	A/0.55	I/0.70	I/0.58

A, active (toxicity predicted); I, inactive (no toxicity predicted).

**Table 10:** Organ toxicity predictions for lead compounds against SARS-CoV-2.

Compound	Toxicity End Points							
	CARD	I	M	CYT	BBB	E	CLIN	N
CHEMBL4472440	I/0.56	A/0.95	I/0.67	I/0.85	A/0.82	A/0.54	I/0.63	I/0.74
CHEMBL1495225	I/0.67	I/0.98	I/0.78	I/0.75	A/0.71	I/0.65	A/0.61	I/0.79
CHEMBL1720210	I/0.55	A/0.62	I/0.64	I/0.72	A/0.68	A/0.58	I/0.6	I/0.65
CHEMBL2177676	I/0.79	I/0.99	I/0.89	I/0.88	A/0.70	I/0.72	A/0.58	I/0.72
CHEMBL4069090	I/0.56	A/0.95	I/0.67	I/0.85	A/0.82	A/0.54	I/0.63	I/0.74

CARD, carcinogenicity; I, immunotoxicity; M, mutagenicity; CYT, cytotoxicity; BBB, blood–brain barrier permeability toxicity; E, ecotoxicity; CLIN, clinical toxicity; N, nutritional toxicity.

**Table 11:** DIGEP-Pred data for drug-induced modifications in expression of genes.

Compound (CHEMBL)	Chemical Structure	I (Pa)	OS (Pa)	A (Pa)	Potential
CHEMBL4472440	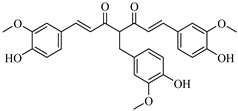	CXCL2 ↓ (0.84)	GCLM ↑ (0.89), SOD3 ↑ (0.85), NQO1 ↑ (0.82), GCLC ↑ (0.78)	TNFRSF10B ↓ (0.82)	Anti-inflammatory, antioxidant
CHEMBL1495225	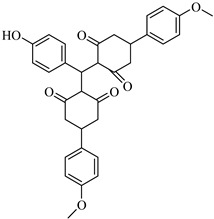	CCL5 ↓ (0.70)	GAS6 ↑ (0.76),	—	Anti-inflammatory, possible antioxidant effect, pro-regenerative
CHEMBL1720210	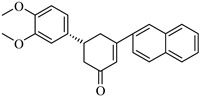	CXCL2 ↓ (0.76), PLCG2 ↓ (0.74)	GAS6 ↑ (0.73)	PARP2 ↓ (0.72)	Anti-inflammatory, antioxidant, pro-regenerative
CHEMBL2177676	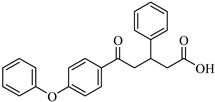	None (no Pa > 0.7)	—	—	—
CHEMBL4069090	** 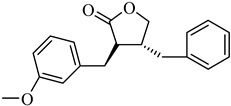 **	None (no Pa > 0.7)	—	CASP2 ↑ (0.69)	Pro-regenerative, potential apoptosis modulation

I, inflammation; Pa, probability active; OS: oxidative stress-related genes; A: apoptosis-related genes; ↑, upregulated; ↓, downregulated.

## Data Availability

The raw data supporting the conclusions of this article will be made available by the authors upon request.

## Abbreviations

The following abbreviations are used in this manuscript:

COVID-19	Coronavirus disease 2019
SARS-CoV-2	Severe acute respiratory syndrome coronavirus 2
ACE2	Angiotensin-converting enzyme 2
3CLpro	3-Chymotrypsin-like protease
PLpro	Papain-like protease
ORF	Open reading frame
nsps	Non-structural proteins
FP2	Fingerprint type 2
SMILES	Simplified molecular input line entry system
PDB	Protein Data Bank
PDBQT	Protein Data Bank, partial charge (q), and atom type (t) format
ADME	Absorption, distribution, metabolism, and excretion
RMSD	Root-mean-square deviation
TPSA	Topological polar surface area
DIGEP-Pred	Drug-induced gene expression profile prediction
Pa	Probability active
